# The Mbeya Antimicrobial Stewardship Team: Implementing Antimicrobial Stewardship at a Zonal-Level Hospital in Southern Tanzania

**DOI:** 10.3390/pharmacy8020107

**Published:** 2020-06-24

**Authors:** Jeffrey W. Hall, Jeannette Bouchard, P. Brandon Bookstaver, Matthew S. Haldeman, Peter Kishimbo, Godlove Mbwanji, Issakwisa Mwakyula, Davance Mwasomola, Megan Seddon, Mark Shaffer, Stephanie C. Shealy, Anthony Nsojo

**Affiliations:** 1 School of Medicine, University of South Carolina, Columbia, SC 29209 USA; Matthew.haldeman@uscmed.sc.edu (M.S.H.); mark.shaffer@uscmed.sc.edu (M.S.); 2 College of Pharmacy, University of South Carolina, Columbia, SC 29208, USA; jbouchar@cop.sc.edu (J.B.); bookstaver@cop.sc.edu (P.B.B.); shealysc@email.sc.edu (S.C.S.); 3 Mbeya Zonal Referral Hospital, Mbeya PO Box 419, Tanzania; pkishimbo@yahoo.com (P.K.); mbwanjigf@yahoo.co.uk (G.M.); ozzanne@gmail.com (I.M.); mwasodav04@gmail.com (D.M.); nsojoa@yahoo.com (A.N.); 4 Sarasota Memorial Health Care System, Sarasota, FL 34239, USA; megan-seddon@smh.com

**Keywords:** antimicrobial stewardship, antimicrobial resistance, Low to Middle Income Countries (LMIC), East Africa, Tanzania, global health

## Abstract

**Background:** In 2017, Mbeya Zonal Referral Hospital (MZRH) and the University of South Carolina (UofSC) agreed to collaboratively strengthen antimicrobial prescribing in the southern highlands of Tanzania and train a new generation of clinicians in responsible antimicrobial use. **Methods:** Key stakeholders and participants were identified and the Mbeya Antimicrobial Stewardship Team (MAST) was created. The team identified assets brought by the collaborators, and four investigations of baseline needs were developed. These investigations included (a) a baseline clinician survey regarding antimicrobial resistance and stewardship, (b) a serial chart review of inpatient antimicrobial prescribing practices, (c) an investigation of antimicrobial resistance rates using existing isolates at the MZRH laboratory, and (d) a survey of antimicrobial availability at community pharmacies in the city. **Results:** 91% of physicians believe antimicrobial resistance is problem in Tanzania, although only 29% of physicians were familiar with the term “antimicrobial stewardship”. *Escherichia coli* isolates had resistance rates of over 60% to the commonly used agents ciprofloxacin, trimethoprim-sulfamethoxazole, and ceftriaxone. Thirteen out of 14 community pharmacies offered over-the-counter antibiotics for upper respiratory symptoms. **Conclusions:** International antimicrobial stewardship collaborations can successfully identify opportunities and needs. Evaluating the team’s efforts to improve patient outcomes will be essential.

## 1. Introduction

Low and middle-income countries (LMICs) shoulder a disproportionate share of many global health problems, including antimicrobial resistance (AMR) [1]. Many LMICs have high rates of routine antibiotic use without a prescription or clinician oversight, and clinicians in all countries often prescribe antibiotics unnecessarily [2]. This problem of antibiotic overuse and the associated specter of rising AMR led the World Health Organization (WHO) to publish a Global Action Plan on Antimicrobial Resistance in 2015 [1,3].

Institutions that have successfully implemented programs to curb inappropriate antimicrobial prescribing have done so through interdisciplinary clinician education, protocol development, and ongoing reviews of guideline adherence [4,5]. Establishment of these antimicrobial stewardship (AMS) interventions has been developed in multiple LMICs [6,7,8,9,10]. Specifically in African nations, AMS implementation has been limited but successful in several published reports, although these programs have additional challenges [11,12,13,14,15,16,17,18,19,20]. A recent global call for collaborative stewardship efforts was made to develop programmatic AMS across LMICs [21].

The University of South Carolina (UofSC) and the Mbeya Zonal Referral Hospital (MZRH) have had collaborative educational programs since 2012. In August 2017, at an on-site visit at MZRH, several staff of these two institutions discussed the creation of an AMS program at MZRH using experiences from the established stewardship program at UofSC and Prisma Health Midlands, the partnering hospital system in Columbia, South Carolina, USA. This article discusses the development of this program, initial results, and lessons learned.

## 2. Materials and Methods 

### 2.1. Partnership Development: Asset Mapping

MZRH is the tertiary referral site for a population of over eight million people, 16% of the country’s population, living in the Southern Highlands Zone of Tanzania [22]. AMR was a preexisting concern of hospital leadership at MZRH, and they actively sought a partnership to work on the problem collaboratively. As a tertiary care center associated with a medical school, it is well suited to implement projects that influence care throughout the region. The hospital’s director of Research and Education is a microbiologist by training and had collected two years of culture data for review and compilation. In addition, the MZRH laboratory had large climate-controlled freezers containing an inventory of many blood and urine isolates from previous years, making them available for further testing. The Prisma Health Midlands, affiliated hospital system of UofSC, stewardship program has been recognized as an Infectious Diseases Society of America (IDSA) Center of Excellence since 2019 [23]. Additionally, the UofSC hosts an infectious diseases pharmacy residency and clinical fellowship program that has significant experience in AMS both in hospital and community settings [24]. The program facilitates a global health experience with MZRH for 4–8 weeks annually. Finally, the UofSC Department of Family and Preventive medicine hosts a Global Health fellowship that allows a clinical faculty member up to 6 months living in Mbeya over two years to facilitate collaborations between the two institutions [25]. UofSC and MZRH have had collaborative projects through this program since 2012.

### 2.2. Partnership Development: Needs Assessment

Tanzania is known to have high rates of ESBL-producing Enterobacteriaceae and beta-lactam resistant *Streptococcus pneumoniae* [26,27,28]. However, no known study has focused on AMR rates in the Mbeya region. There are reasons to suspect an altered AMR pattern in the Southern Highlands region of Tanzania; it borders two neighboring countries with relatively high cross-border traffic, and it also has some of the highest rates of HIV in the nation [29]. As for antimicrobial use in the region, there had never been a systematic investigation of antibiotic prescribing at the hospital or community levels in Mbeya. Several of the authors noted anecdotally that MZRH wrestles with issues common to many LMICs, including medication access difficulties, lack of local guidelines, and inconsistently performed diagnostics. 

### 2.3. Creation of the Antimicrobial Stewardship Team

**Figure 1:**
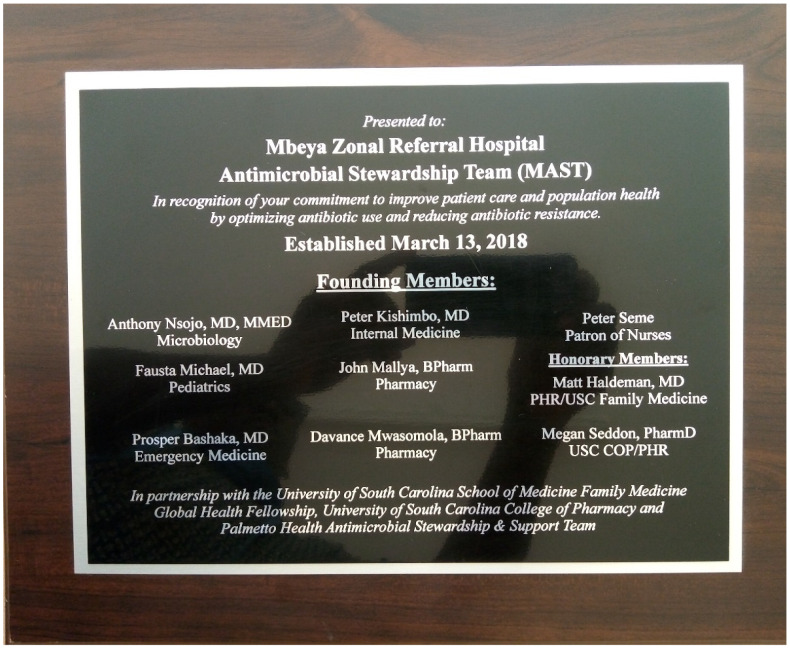
Mbeya Zonal Referral Hospital Antimicrobial Stewardship Team (MAST) established 13 March 2018.

After recognizing the mutual interest in strengthening AMS efforts at MZRH and identifying the assets shared between the two institutions, a partnership was agreed upon to develop an AMS team, including representatives from MZRH pharmacy, nursing, physician and administrative departments, along with pharmacy and physician members from UofSC. This group was titled the Mbeya Zonal Referral Hospital Antimicrobial Stewardship Team (MAST) (Figure 1). The UofSC global health fellow for the years of 2017–2019 focused efforts on developing this project. Teleconferences and video meetings were scheduled connecting the team from both institutions more frequently than travel allowed.

### 2.4. MAST: Initial Investigations

The first task of the newly formed MAST was to formally investigate the status of current AMR and antimicrobial use in the region. Some countries and institutions might view this kind of internal retrospective evaluation as a quality improvement process rather than as human subjects research, but not every nation recognizes such a distinction. Ethical review for this study was completed in a step-wise fashion, according to the process established by Tanzania’s National Institute of Medical Research (NIMR). First, the study proposal was submitted for review to the IRB at MZRH, with the assistance of Tanzanian study collaborators. Once this approval was granted, the authors then completed submission for ethical review at NIMR headquarters in Dar es Salaam, Tanzania. The proposal was also submitted to the IRB at UofSC who evaluated the program through the process of facilitated review. As required by NIMR, the authors also obtained permission to publish from NIMR prior to any manuscript submissions for publication. The multi-step process took approximately 4 months. 

**Figure 2:**
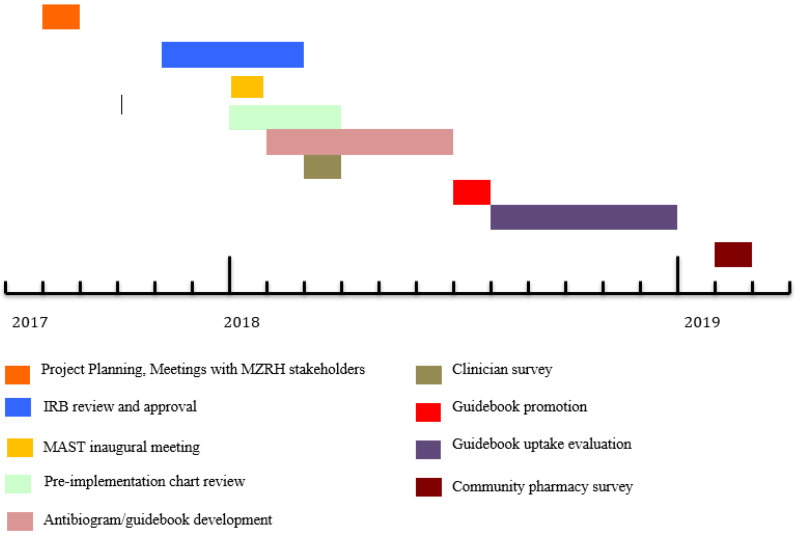
Timeline of Mbeya Antimicrobial Stewardship Team (MAST) activities.

When IRB review was granted, four baseline assessments were performed: a survey of MZRH clinician perceptions of AMR, a chart review to investigate clinician antibiotic utilization patterns in the inpatient setting at MZRH, an assessment of local AMR patterns using MZRH culture data, and a survey of over-the-counter availability of antibiotics at local pharmacies. Refer to Figure 2 for a timeline of implementation activities.

- **Baseline Clinician Survey:** A 16-question clinical survey regarding local AMR patterns and AMS concepts was administered to MZRH clinicians of all cadres in the departments of Internal Medicine and Pediatrics. The survey assessed clinicians’ awareness of local AMR patterns, confidence in making empirical antibiotic treatment decisions, perceived barriers to MZRH patients receiving optimal antibiotic therapy, and familiarity with the concept of AMS. Of the 16 questions, 12 were based on a 5-point Likert scale. Two were single-answer multiple choice, and 1 was a multiple choice in which more than one answer could be selected. One additional question was a ranking question in which respondents were asked to rank items in order from 1 through 6, with 1 being the most important factor and 6 being the least. The survey was anonymous, but respondents were asked to provide their designations (“intern”, “registrar”, “specialist”, etc.)
- **Chart Review of Antimicrobial Prescribing Practices:** A retrospective chart review assessed baseline inpatient antibiotic utilization patterns at MZRH. Beginning with 1 January 2018, the authors chronologically reviewed all adult inpatient medical records for the MZRH adult male and female medical wards until 100 charts involving antibiotic therapy were included. Data points collected included antibiotic-specific metrics, including antibiotic name, indication, days of therapy prescribed, and days of therapy administered. Other data points collected included patient demographics, infection-pertinent comorbidities, length of stay, 30-day readmission, in-house mortality, payer status, and culture utilization. Additional calculated metrics included frequency of antibiotic course completion, antibiotic use per infectious indication, and concordance with national antibiotic guidelines, specifically the Tanzania Standard Treatment Guidelines and National Essential Medicines List, 5th edition [30]. A detailed description of the methods for this investigation is published elsewhere [22].
- **Local Antimicrobial Resistance Patterns:** To assess AMR patterns, the MAST evaluated MZRH culture and resistance data, as cataloged by the MZRH microbiology department over 2 years prior to this study’s initiation. Cultures included specimens from multiple body sites. All of the positive cultures were speciated using manual techniques, and antibiotic susceptibility was determined using Kirby–Bauer (KB) disk diffusion method. To ensure reliability of the KB methodology, a microbiologist randomly selected 25 Enterobacteriaceae isolates (comprising *Escherichia coli* and *Klebsiella species*) and performed a second independent evaluation of resistance to these agents using E-tests.
- **Community pharmacy survey:** Finally, we surveyed local pharmacies in the city of Mbeya to assess the availability of antibiotics without a prescription (“over-the-counter”). Two teams of 2 authors each presented to 15 different local Mbeya pharmacies complaining of nonspecific upper respiratory symptoms. The pharmacy name, location, designation of the pharmacy employee (“pharmacy technician”, “pharmacist”, etc.), antibiotics offered, and selling prices were recorded.

## 3. Results

### 3.1. Baseline Clinician Survey

There were 45 respondents to the baseline clinician survey, and 78% have been in practice for ≤ 5 years. Overall, 91% of respondents feel that AMR is a problem nationally, while 82% believe it is also a problem locally at MZRH. In contrast, over 30% of respondents were not aware of AMR patterns either nationally or locally. The overwhelming majority of respondents were confident in their ability to select appropriate empirical and definitive antibiotic choices, interpret microbiology results, and discuss or teach antibiotic therapy with a patient or colleague, respectively. Most respondents (78%) reported they use published guidelines to aid in empirical antibiotic decisions, while only 8% use peer-reviewed primary literature. Only 29% were familiar with the term “antimicrobial stewardship”. Full results are available in Table 1. Due to the relatively small numbers in the survey, we did not perform statistical calculations based on clinician subgroupings. However, more experienced clinicians did generally report greater familiarity with AMS concepts. For example, 6 out of 7 attending physicians indicated they were familiar with the term “Antimicrobial Stewardship”, while only 7 of 38 clinicians at earlier stages of their career responded in that way. 

### 3.2. Chart Review of Antimicrobial Prescribing Practices

The structured analysis of antimicrobial ordering and administration confirmed anecdotal suspicions of frequent variations from national guidelines for empiric therapy. Empiric treatments for the six most common indications were inconsistent with national guidelines more than 50% of the time. Two-thirds of prescribed antimicrobial courses in the hospital setting were not completed for various reasons. Full results are published elsewhere [31].

### 3.3. Local Antimicrobial Resistance Patterns

**Figure 3:**
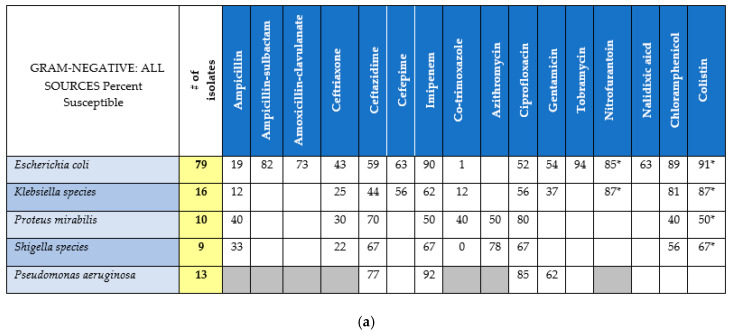

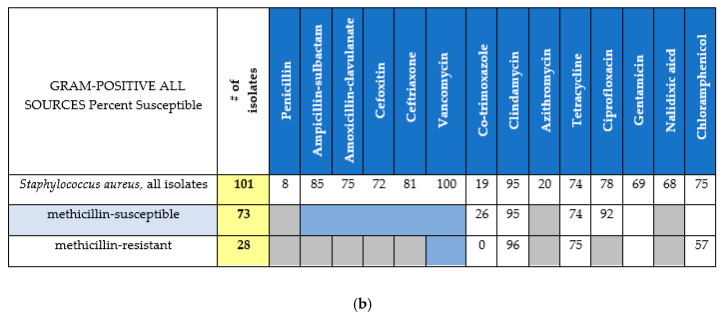
(**a**) Gram-negative Antibiogram, all sources. (**b**) Gram-positive Antibiogram, all sources. Blank boxes represent unavailable data. Gray boxes represent intrinsic resistance or not recommended. Blue boxes represent deduced/assumed susceptibility, although not specifically tested. * Only tested against select isolates.

**Figure 4:**
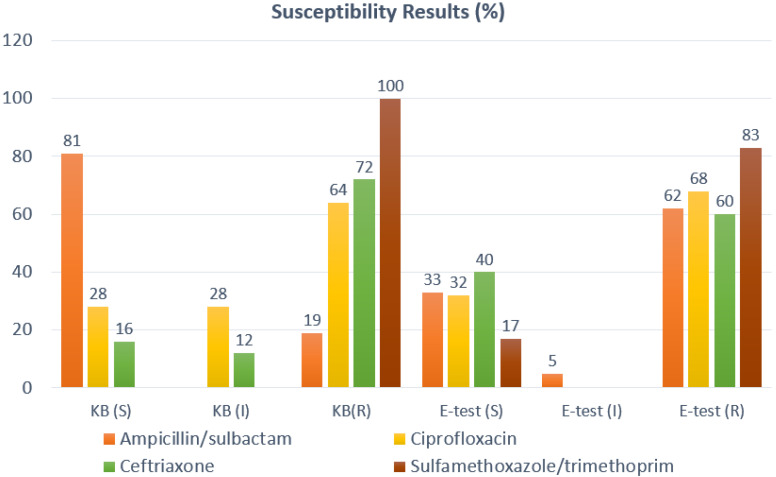
Results of concordance among Kirby–Bauer and E-test results.

The culture data was collated into an MZRH antibiogram, (Figure 3a,b) consisting of 5 Gram-negative species (127 isolates) and one Gram-positive species (*Staphylococcus aureus*, 101 isolates). The antibiogram summarized bacterial species’ susceptibility to commonly used local antibiotics. Due to manual testing, there is some unexpected variability in reporting (e.g., amoxicillin/clavulanate versus ampicillin/sulbactam differences for MSSA). One notable finding was the exceptionally high resistance rates (>95%) of *E. coli* to trimethoprim-sulfamethoxazole. Confirmatory testing did reveal some discordance between E-test and KB methodologies, particularly with regards to ampicillin/sulbactam susceptibilities. However, results were fairly concordant with regards to ceftriaxone, ciprofloxacin, and trimethoprim-sulfamethoxazole (Figure 4). Due to low number of isolates, inferential statistics were not performed.

### 3.4. Community Pharmacy Survey

Also consistent with anecdotal impressions and published reports, antimicrobials are widely available over-the-counter within the Mbeya community [32,33]. Of the 14 community pharmacies surveyed, 13 provided antibiotics for complaints consistent with Upper Respiratory Infection (URI) symptoms without a prescription required. Antibiotic regimens for the standard URI script ranged from 3–7 days and included a wide variety of agents (Table 2).

## 4. MAST: Analysis to Implementation

### Antimicrobial Guidebook

**Figure 5:**
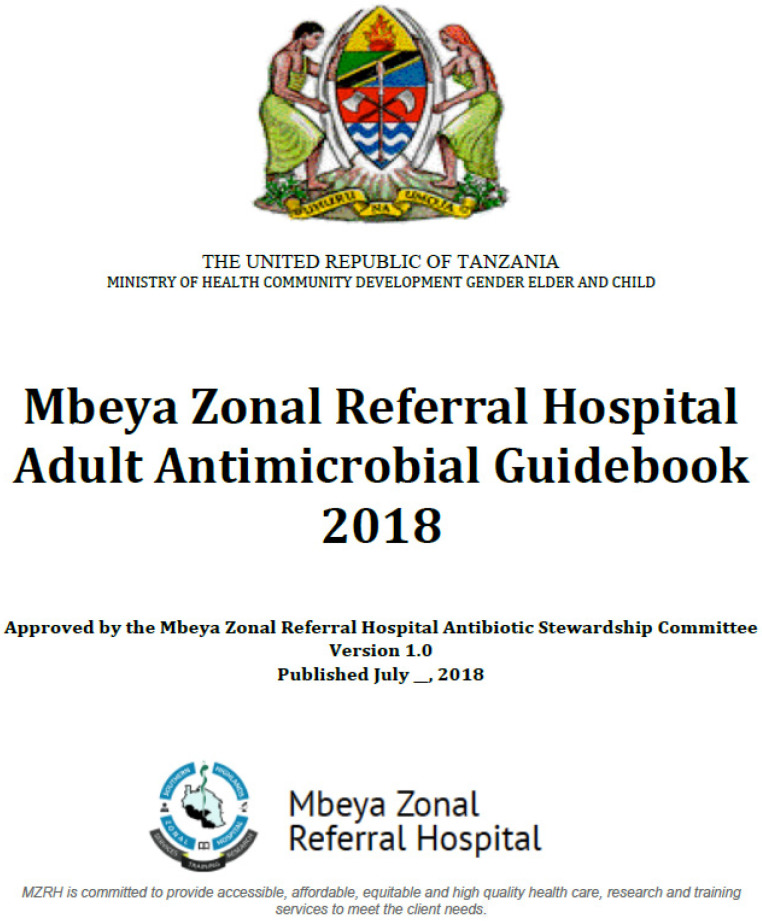
Cover of the Mbeya Zonal Referral Hospital Adult Antimicrobial Guidebook 2018 (1st edition). [Note: Full guidebook available upon request].

Armed with information on local AMR patterns, local prescribing patterns, and clinician perceptions on AMS, the MAST believed this data could inform a local guide to complement the Tanzanian national guidelines for empirical therapy. These guidelines are widely available and offer recommendations for empirical therapy based on suspected or proven source of infection [30]. However, these guidelines do not fully account for variable patient populations, local resistance patterns, or local medication supply chains. The MAST believed that some of these difficulties could contribute to the relatively low adherence of empiric antimicrobial regimens to the national guidelines and frequently inappropriate truncations of therapy while in the hospital. Additionally, the MAST felt that a brief local guidebook could be made available through electronic media (e.g., WhatsApp™) to all house staff and to improve uptake by the medical staff. This particular electronic platform was used to distribute the guidebook because it is already widely available among the clinicians of MZRH on their personal devices. It does, however, require the clinicians to use their personal data plan to download the document. The MZRH Antibiotic Guidebook 1st edition would focus on the most common bacterial infectious diseases encountered. Diseases such as malaria, HIV, or tuberculosis, while common, were considered to be outside the scope of the guidebook. The final list of disease states included skin and soft tissue infections, cystitis, pyelonephritis, meningoencephalitis, bacterial pneumonia, enteritis/colitis. A special section on general management of sepsis was added upon request. Decisions regarding empirical drug choice weighed each of the following: known local resistance patterns, consistency of drug availability, national guideline recommendations, other evidence-based recommendations, and clinician experience (Figure 5, cover of guidebook only; full guidebook is available upon request).

Once the MZRH antimicrobial guidebook was complete and approved by the MAST, the MZRH hospital administration and medical staff, it was promoted among all MZRH clinical staff as a hospital standard-of-care. The MAST first presented the guidebook to MZRH interns, registrars, and attending physicians at morning report for the departments of internal medicine, pediatrics, and obstetrics/gynecology (OBGYN). Subsequently, we presented the guidebook again to the hospital-wide medical staff at MZRH’s weekly staff education meeting. The guidebook was also emphasized by several authors on daily internal medicine teaching rounds, where its application to patient care was demonstrated at the bedside. An electronic copy of the guidebook was distributed to all MZRH interns and registrars in PDF format via WhatsApp™ Messenger. Paper copies of the guidebook were posted at the nurses’ station of each MZRH ward, and available nurses and students of all types were educated on the guidebook’s utility at that time.

Over the next several months, the authors evaluated uptake and usage of the guidebook at MZRH via several methods. First, certain MZRH interns and registrars were designated as key respondents, and the authors inquired about ongoing guidebook utilization through regular text message communications with these clinicians. Next, regular videoconferences were held between UofSC clinicians and the MAST to discuss current guidebook utilization and future promotional activities.

## 5. Discussion

As has been detailed above the MZRH and UofSC partnership has been a fruitful endeavor over the past several years. Here we will attempt to synthesize the results from our initial projects as well as review successful strategies, lessons learned and remaining challenges.

### 5.1. Discussion of Results

While the term “Antimicrobial Stewardship” is not widely known by junior Tanzanian physicians, the problem of AMR is clearly recognized. There was therefore little need to persuade the new house staff about the need for responsible antimicrobial therapy. It was perhaps surprising, however, to note how empirical treatment infrequently fully aligned with national guidelines. While nearly two-thirds of patients were initiated on the recommended agents, only 15% were able to complete the required therapy [31]. Empirical recommendations and durations are of paramount importance, because in our chart review of inpatient prescribing, only 15% of patients had diagnostic testing. Diagnostic testing will often be considered an unaffordable luxury as long as financial resources remain scarce at MZRH. In the meantime, ensuring patients reliably receive appropriate durations of appropriate antimicrobials should improve outcomes. 

We expected to find a degree of AMR in the isolates available at the MZRH lab but did not expect such substantial resistance to trimethoprim/sulfamethoxaxole. This could be the result of widespread availability of antibiotics in the community, *Pneumocystis jirovecii* prophylaxis in the HIV-infected population, or the historical use of sulfa-based antimalarials. This finding demonstrates the need for local AMR data to guide the empirical recommendations. However, the significant discordance between KB and E-test methodologies for certain antimicrobial susceptibilities in the MZRH sample of isolates highlights some difficulties in obtaining reliable resistance data in LMIC settings.

### 5.2. Successful Strategies 

One dominant theme in productive global health work is to work in a spirit of collaboration and partnership, rather than of one-sided mentorship. This program was built on 5 years of the existing partnership between our institutions with the strong interest of local leadership in AMS guiding project development. All aspects of the project were performed in collaboration with careful thought and consideration of local and national standards of care and local resource availability. A broad level of support across each institution proved very helpful in developing the multi-modal evaluations needed to start an AMS effort in Southern Tanzania. We felt we were better able to inform our investigations and tailor our interventions to the local situations because we had input from a wide range of colleagues. 

Having multiple supporting collaborators across the organizations has been critical, but this project has required a specific champion at each institution to keep the project moving forward. The local champions have been the ones responsible for organizing meetings, setting agendas, and following up on individual tasks. Without an individual taking responsibility for shepherding the team, projects and team meetings would stumble. 

Within a spirit of broad-based partnership, some successes came by not only assessing needs and identifying gaps in care, but also recognizing institutional strengths and resources. For example, by using existing MZRH data and isolates already collected, we were able to reach conclusions about local resistance much more quickly and economically than if dependent on prospective collection alone. From the UofSC side, contributing the time and expertise of our Global Health Fellow provided the program with a dedicated collaborator who could spend time in both sites and initiate projects. Incorporating a global rotation into the Infectious Diseases pharmacy residents’ academic schedule also allowed these pharmacists time to substantially contribute to the program. Building from existing institutional strengths has helped to keep costs low as no external funding was used for the initiation of this program. The lack of dependence on external grant funding to start up the AMS collaboration should bode well for its sustainability.

Another practical strategy is the use of virtual means to conduct meetings and write protocols and papers collaboratively across time zones. Travel back and forth between South Carolina and Mbeya is time consuming and costly. A collaborative stewardship team requires a relatively small increments of time invested over a longer period. Recurring international in-person meetings are not possible, but recurring virtual meetings are much more plausible.

While our overarching long-term goal has been to improve patient outcomes through improved antimicrobial stewardship, short-term victories have also been encouraging to those involved. All US and Tanzanian partners grew through this process in terms of international collaboration, academic leadership, and research and publication experience.

### 5.3. Lessons Learned

As with any partnership, several challenges remain from practical human issues to larger scale economic and systemic concerns. Travel to Tanzania or South Carolina by respective institutions is limited to once or twice a year for this reason. Having existing personal relationships from prior projects has been an important foundation in building the program. When the program was first introduced, extensive time and resources were required from both institutions for implementation and education. Continued education to the new health care staff at MZRH has relied heavily on daily rounds by MZRH program partners and annual visits from UofSC partners. Local investment in this collaboration has become more important for providing continual education and sustaining the program.

Thus, more frequent teleconferences and video meetings with MAST and UofSC were implemented to assist with continued education and problem-solving any barriers that were presenting themselves throughout the month. Virtual meetings have been an essential component to this program, despite they too have significant practical challenges. The very clinicians and pharmacists that are best suited to provide “on the ground” insight at the meetings are also those most likely to be called away to other duties. Differing time zones and clinical schedules add additional complexity to arranging meetings without infringing on other responsibilities. We have found that most work happens asynchronously through email or other means, with organized video or in-person meetings relatively rare and focused on relationship building and items that require real-time discussion. 

Academic development has been a real strength of our collaboration, although funding for collaborators for presenting findings at scientific meetings has been limited to what host institutions have been able to provide within routine budgets. Since LMICs run on smaller budgets, they have had fewer opportunities to present their ideas to a wider audience.

Working in resource-limited environments presents ethical challenges when an ideal local, affordable, and available treatment may not always be possible. The MAST team cannot solve these economic realities independently, but by collecting and publishing data on resistance rates, we feel we provide an avenue to advocate for access on the national level. A short-term strategy could include dual guidelines that include both optimal treatments along with more affordable, albeit suboptimal options. Such dual guidelines may be very practical but are a very sensitive topic in that they expose disparities in care.

### 5.4. Future Directions

MZRH is a teaching hospital, with a new cohort of clinicians arriving every year. This is a great opportunity to influence a new generation of physicians on responsible antimicrobial use, but it comes with the great challenge of educating a new group every single year. Building a culture of responsible antimicrobial use is not a process that will be completed in two years. The antimicrobial guidebook is an exciting new step, although its effectiveness to alter prescribing habits or improve patient outcomes remains to be seen. Most institutions that have seen demonstrable changes in these indicators did release institution specific guidelines, but also had more effective real-time tracking mechanisms and more rigorous ways to encourage change. There are plans to investigate the impact of initial strategies on guideline compliance and to develop and implement additional interventional stewardship activities, such as prospective audit and feedback.

Sustainability of the program requires the use of readily available data to drive decisions making. One data point commonly used in the field of AMS is antimicrobial consumption data. Variability in supply and variability in sources of antibiotics at MZRH has meant that, so far, no reliable and easily repeatable estimate of antimicrobial consumption at the hospital has been identified. Without such large-scale assessments, prescribing habits require serial chart reviews, which are labor-intensive and time-consuming. Developing a low-cost, high-fidelity mechanism to assess habits will be integral to the success of the program.

One ideal method to increase AMS sustainability and timeliness of intervention uptake is to develop a more formal AMS training program in partnership between the two collaborating institutions. A very important aspect of this success would be the ability to bring MZRH colleagues to the United States to experience AMS at UofSC and Prisma Health Midlands. This strategy has been successfully utilized by others [34]. Facilitating collaborations with similar groups in East Africa or other LMICs would also bring new insights into addressing the practical problems of AMS in these regions [20].

## 6. Conclusions

The partnership between the MAST and the UofSC has had some real successes. It has helped introduce the concept of AMS to a new generation of learners in East Africa. It has also provided some specific data on prescribing habits in the region, availability of antimicrobials in the community, and illustrations of difficulties with adhering to guidelines in an economically constrained region. The partnership has also been successful in cultivating academic productivity in newer clinical faculty with limited prior experience in disseminating research, and it has helped provide a platform for the exchange of ideas and experiences between institutions.

The work is still challenging. Inappropriate use of antibiotics is a stubborn problem throughout the world, and this remains true at both of our institutions. Clearly, such partnerships remain critical. Antimicrobial resistance will inevitably become an increasing problem in LMICs as antimicrobials become more available in these regions. Developing strong AMS teams at teaching hospitals across the globe is a critical strategy to address this problem in the coming years.

## Figures and Tables

**Table 1:** Baseline Clinician Survey Results *.

Question	1	2	3	4	5
Q1: I believe antibiotic resistance is a problem nationally in Tanzania.	4.5%	2.3%	2.3%	36.4%	**54.5%**
Q2: I believe antibiotic resistance is a problem locally at Mbeya Zonal Hospital.	9.1%	2.3%	6.8%	**52.3%**	29.5%
Q5: I believe my patients receive antibiotics in a timely fashion following suspected or confirmed infectious diagnoses.	6.7%	15.6%	15.6%	**33.3%**	28.9%
Q6: I am confident in my ability to select appropriate empirical antibiotic therapy based on a given diagnosis.	2.2%	4.4%	6.7%	33.3%	**53.5%**
Q7: I am confident in my ability to accurately interpret culture and susceptibility reports.	0.0%	2.3%	4.5%	18.2%	**75.0%**
Q8: I am confident in my ability to determine appropriate definitive antibiotic therapy based on available diagnostic reports.	0.0%	2.2%	2.2%	33.3%	**62.2%**
Q9: I am confident in my ability to discuss antibiotic therapy with a patient.	2.2%	2.2%	8.9%	37.8%	**48.9%**
Q10: I am confident in my ability to discuss or teach antibiotic therapy principles to a peer.	4.4%	0.0%	8.9%	31.1%	**55.6%**
Q11: I am aware of antibiotic resistance patterns nationally.	8.9%	13.3%	13.3%	**46.7%**	17.8%
Q12: I am aware of antibiotic resistance patterns at Mbeya Zonal Hospital.	17.8%	8.9%	13.3%	**51.1%**	8.9%
Q13: I am familiar with the term antimicrobial stewardship.	**31.8%**	15.9%	22.7%	13.6%	15.9%
Q14: I am familiar with the approximate costs of commonly used antibiotics that hospitalized patients are prescribed.	6.7%	8.9%	13.3%	**55.6%**	15.6%

* Not all respondents answered every question. Due to rounding, percentages may not add up to exactly 100%. 5 = Strongly Agree; 4 = Somewhat Agree; 3 = Neutral; 2 = Somewhat Disagree; 1 = Strongly Disagree. Bold number: the most frequent response.

**Table 2:** Antimicrobials provided by community pharmacies for upper-respiratory tract infection symptoms.

Pharmacy Number	Antibiotic Regimen	Cost (TSH)
1	Amoxicillin 500 mg 3× daily × 3 days	2000
2	Azithromycin 500 mg daily × 3 days	2550
3	Ampicillin/cloxacillin 250/250 mg 3× daily × 5 days	2000
4	Azithromycin 500 mg daily × 3 days	16,400
5	Sulfamethoxazole/Trimethoprim 2 SS tablets 2× daily × 7 days	3000
6	Amoxicillin/Clavulanate 625 mg 2× daily × 7 days	32,000
7	Amoxicillin 500 mg 3× daily × 5 days	30,000
8	Ampicillin/Cloxacillin 250/250 mg 3× daily × 5 days	3000
9	Cephalexin 250 mg 2 tablets 3× daily × 5 days	4000
10	Erythromycin 250 mg 2 tablets 3 × 5 days	3000
11	Lomefloxacin daily × 5 days	3000
12	Cefpodoxime 100 mg 2× daily × 5 days	25,000
13	Cefuroxime 250 mg BID × 5 days	18,000
14	None Offered	N/A
